# Comparison of conventional culture and automated blood culture system for microbiologic diagnosis of pleural infection

**DOI:** 10.1128/spectrum.00352-25

**Published:** 2025-06-12

**Authors:** Young Ho Lee, Min Seo Kang, Jinyoung Yang, Si-Ho Kim, Jae-Hoon Ko, Sun Young Cho, Tae Yeul Kim, Hee Jae Huh, Nam Yong Lee, Cheol-In Kang, Doo Ryeon Chung, Kyong Ran Peck, Kyungmin Huh

**Affiliations:** 1 Division of Infectious Diseases, Department of Internal Medicine, Chung-Ang University Hospital, Chung-Ang University College of Medicine 37974, Seoul, Republic of Korea; 2 Division of Infectious Diseases, Department of Medicine, Samsung Medical Center, Sungkyunkwan University School of Medicine 35019, Seoul, Republic of Korea; 3 Division of Infectious Diseases, Department of Medicine, Samsung Changwon Hospital, Sungkyunkwan University School of Medicine 35019, Changwon, Republic of Korea; 4 Department of Laboratory Medicine and Genetics, Samsung Medical Center, Sungkyunkwan University School of Medicine 35019, Seoul, Republic of Korea; Icahn School of Medicine at Mount Sinai, New York, New York, USA

**Keywords:** pleural infection, conventional culture, automated blood culture

## Abstract

Microbiological diagnosis of pleural infection is often hindered by the low sensitivity of conventional culture. The automated blood culture system (ABCS) has been shown to improve diagnostic sensitivity for the culture of non-blood specimens. This study examined the additional diagnostic benefit of ABCS against conventional bacterial culture in patients who underwent thoracentesis or percutaneous drainage. Non-duplicate patients whose pleural fluid samples were tested using both conventional culture and ABCS (BACT/ALERT 3D and VIRTUO, bioMerieux) from 2001 through 2021 were included. Cases were excluded if only contaminants such as coagulase-negative staphylococci, *Corynebacterium* species, and *Bacillus* species were identified. Data on culture results, demographics, and laboratory tests were collected. Of the 9,020 patients, 632 patients had positive results in ABCS after excluding those considered contaminants (*n* = 180) or those positive only in conventional culture (*n* = 5). Conventional culture was positive in 302 (47.8%) patients, whereas 330 (52.2%) patients had isolates only from ABCS. Patients with positive results from ABCS alone had lower pleural fluid lactate dehydrogenase and serum C-reactive protein (CRP) and higher pleural fluid glucose and protein. Among organisms isolated from ABCS alone, viridans group streptococci*, Staphylococcus aureus,* and *Klebsiella* spp. were the most common organisms identified. Higher pleural fluid glucose and blood white blood cells and lower CRP were significant factors associated with exclusive ABCS positivity. More than half of the patients whose pleural fluid cultures were positive using ABCS had a negative result on conventional culture. Our results suggest that ABCS might enhance the microbiologic diagnosis of pleural infection.

**IMPORTANCE:**

This study demonstrated that an automated blood culture system (ABCS) has superior sensitivity in pleural infections compared with conventional culture. By comparing both methods in over 9,000 patients, researchers found that ABCS detected bacteria in approximately twice as many cases as conventional culture, especially in patients with a high probability of pleural infection. The findings suggest that ABCS can be a valuable tool in improving the accuracy of diagnosing pleural infections, which could lead to better treatment decisions and patient outcomes.

## INTRODUCTION

Pleural infection is a major cause of morbidity and mortality in adults. Its incidence is increasing in many countries (1–3). Its high morbidity is associated with prolonged hospitalization and substantial post-discharge sequelae, leading to a significant economic burden, with annual costs estimated at $500 million in the United States (4). Furthermore, in the case of empyema, clinical outcomes are notably poor, with 20% of cases requiring surgery and one in five resulting in death within the first year of diagnosis (5). Pleural infection is known to exhibit unfavorable outcomes, especially in the elderly and/or immunocompromised patients (6).

The cornerstone of treating pleural infection lies in appropriate antibiotics and drainage, considering local microbiology policy or guidelines and patterns of antibiotic resistance (7). Identifying the offending organism is crucial, as it can reduce unnecessary broad-spectrum antibiotic usage, minimize drug-related side effects, and decrease antibiotic resistance (8). However, microbiological diagnosis of pleural infection is often challenging due to the low sensitivities of conventional culture methods, with causative organisms remaining unidentified in approximately 40% of the cases (9).

It has been reported that an automated blood culture system (ABCS) can enhance the microbiological diagnostic sensitivity even when non-blood sterile body fluids are used (10–12). A study specifically targeting pleural fluid has reported that diagnostic sensitivity is increased when ABCS is used (13, 14). However, these studies have a limitation of small sample sizes. Thus, we compared the diagnostic yields of conventional culture and ABCS in a large number of patients who underwent thoracentesis or percutaneous drainage of pleural fluid with positive cultures and examined the factors associated with positive cultures in ABCS only.

## MATERIALS AND METHODS

### Study design and patient selection

This was a retrospective, single-center study based on data from the Clinical Data Warehouse DARWIN-C of Samsung Medical Center, a tertiary care hospital located in Seoul, Korea. We initially screened patients who underwent thoracentesis or percutaneous drainage and had pleural fluid samples tested with both conventional culture and ABCS between January 2001 and December 2021. Among these, only patients with microbiologically confirmed pleural infection, defined as the isolation of clinically significant microorganisms from pleural fluid, were included in the final analysis. If pleural fluid tests were performed in duplicate, only the first test was included. We excluded cases from the study when only microorganisms generally considered contaminants, such as coagulase-negative staphylococci, *Corynebacterium* species, and *Bacillus* species, were identified or when microorganisms were isolated only in conventional culture but not in ABCS.

Approval for this study was obtained from the institutional review board (IRB) of Samsung Medical Center (IRB No. 2023-12-072). The requirement for informed consent was waived by the IRB because of the retrospective nature of this study and the analysis using anonymous clinical data.

### Data collection and definition of antimicrobial resistance

Baseline patient characteristics, laboratory results (blood and pleural fluid), and culture results were collected. Pleural fluid parameters, including white blood cells (WBC), protein, pH, glucose, lactate dehydrogenase (LDH), and blood parameters, including WBC, C-reactive protein (CRP), LDH, and protein, were obtained at the time of thoracentesis or percutaneous drainage catheter insertion. Presence of fever was defined as a tympanic temperature of 38°C or higher on the day preceding thoracentesis or percutaneous drainage catheter insertion. Since the upper report limit of WBC in pleural fluid at our center was 1,000 /mm^3^, the pleural fluid WBC count was divided into two groups: 0–500/mm^3^ vs >500/mm^3^.

Culture results and antimicrobial susceptibility test results were also collected. Routine microbiologic test procedure in our center is as follows. Pleural fluid samples were typically divided at the bedside immediately after collection. Whenever possible, approximately 5–10 mL of pleural fluid was inoculated into each aerobic and anaerobic blood culture bottle (for ABCS), and approximately 5–10 mL was placed into a sterile tube for conventional culture. All samples were then promptly transported to the microbiology laboratory for processing. The lower portion of the pleural fluid sample, which was not centrifuged or spun, was aspirated and inoculated for culture. For conventional culture, only solid media were used, specifically blood agar plate, MacConkey agar, and Brucella agar. These plates were incubated under both aerobic and anaerobic conditions at 37°C for up to 7 days. ABCS utilized both aerobic and anaerobic culture bottles that were also incubated at 37°C for up to 7 days before undergoing terminal subculture. No additional growth supplements were used in either conventional culture or ABCS. ABCS included BACT/ALERT 3D and VIRTUO system (bioMérieux, BACT/ALERT 3D was replaced by BACT/ALERT VIRTUO in July 2020). VITEK 2 and VITEK MS (bioMérieux, Marcy-l'Étoile, France, VITEK 2 was replaced by VITEK MS in May 2016) were used for species identification and susceptibility testing. The susceptibility of isolates was determined according to the Clinical and Laboratory Standards Institute guidelines (15). Furthermore, we utilized the 2021 Centers for Disease Control and Prevention antimicrobial resistance phenotype definitions to classify resistance for aminoglycoside (AGR), fluoroquinolone (FQR), extended-spectrum cephalosporin-resistant (ESCR), carbapenem (CR), and multidrug-resistance (MDR). To align with other definitions, intermediate susceptibility was categorized as resistant in our study (16). To assess the concordance between the two methods, we examined not only the positivity of culture but also the agreement in species identification and the number of isolated species.

### Subgroup and post-hoc analyses

We conducted subgroup analyses for two groups based on whether they satisfied the criteria of pleural fluid pH ≤7.2 or pleural fluid glucose <60 mg/dL to examine diagnostic characteristics in the patients with a higher probability of pleural infection or complicated parapneumonic effusion according to the British Thoracic Society guidelines (17). Also, the results from BACT/ALERT 3D and VIRTUO were compared separately against conventional culture to examine the diagnostic characteristics of the two ABCS systems.

### Statistical analysis

Discrete data are described as numbers (percentages), and continuous data are described as mean and standard deviation or median and interquartile range (IQR) according to sample size. For further analysis and comparisons, the Pearson *χ*² test was used for categorical variables, and Student’s *t*-test was used for continuous variables when comparing different groups. All analyses were two-tailed, and a *P*-value < 0.05 was considered statistically significant.

Multiple logistic regression analysis was conducted with positive culture in ABCS alone as the dependent variable to identify patient groups that could benefit from ABCS. All statistical analyses were performed using IBM SPSS Statistics (Version 28.0, IMB Corp., Armonk, NY, USA).

## RESULTS

### Characteristics of patients

**Fig 1:**
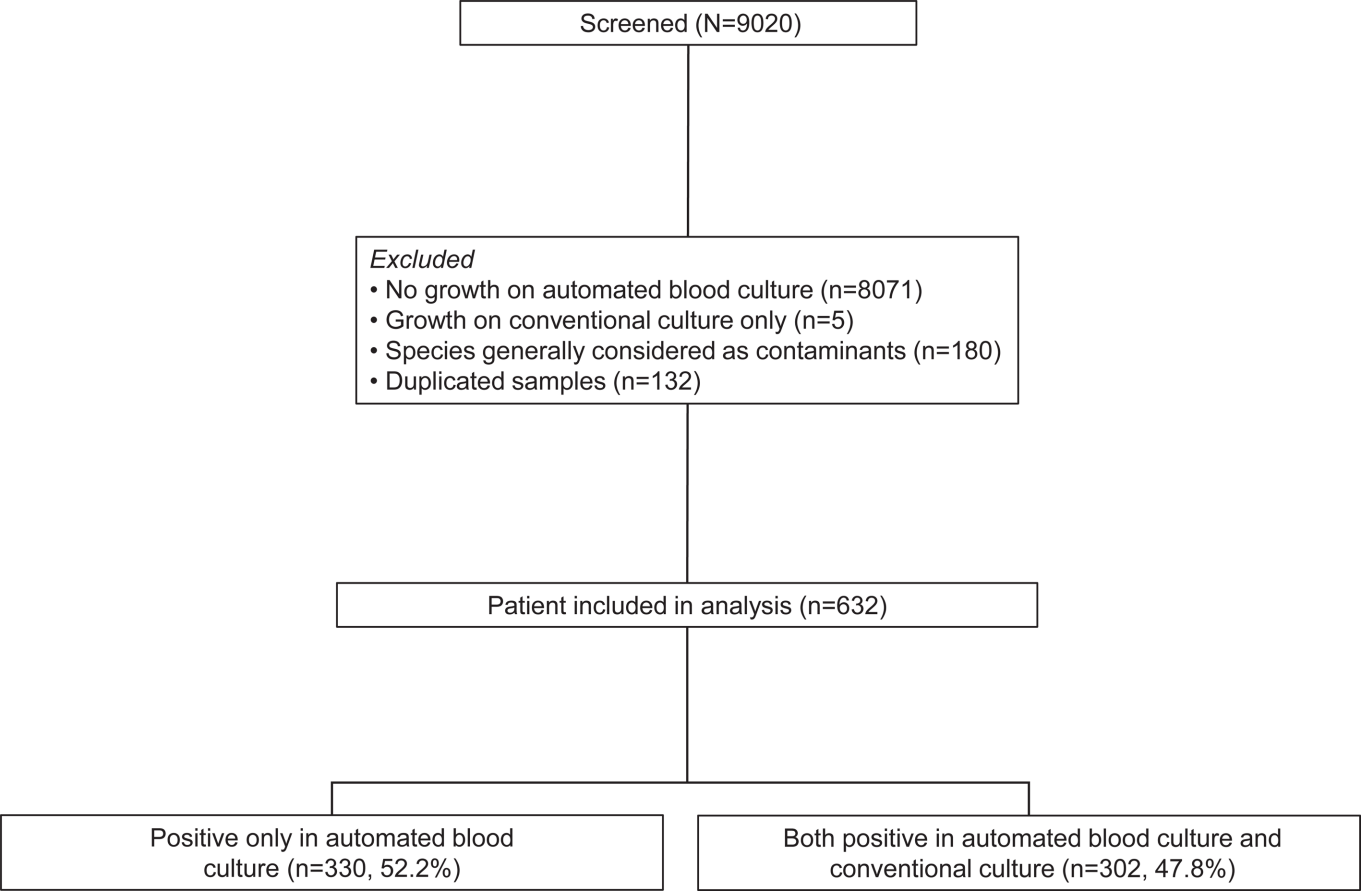
Study flow chart.

A total of 9,020 patients underwent thoracentesis or percutaneous drainage from January 2001 to December 2021. After excluding 8,071 patients where microorganisms were not identified from ABCS, 180 patients from whom probable contaminants were isolated, and five cases in which microorganisms were only isolated from conventional culture, 632 patients were included in the study (Fig. 1). The five cases with growth exclusively in conventional culture were excluded from the main comparative analysis due to the rarity of such occurrences; their clinical characteristics are presented separately in Table S1. Among 632 patients included in the analysis, 330 (52.2%) patients had microorganisms identified only with ABCS, whereas 302 (47.8%) patients had microorganisms identified with both methods.

Characteristics of patients based on bacterial culture results are presented in Table 1. There was no significant difference in gender between the two groups, although patients with positive culture only in ABCS were slightly older. Pleural fluid WBC count and LDH were significantly higher in patients with positive conventional culture and ABCS than in those with positive ABCS alone. In contrast, pleural fluid glucose, pH, and protein were significantly lower in patients with positive conventional culture and ABCS. There were no significant differences in blood chemistry except for CRP, which was significantly higher in patients with positive conventional culture and ABCS. There were no significant differences in the presence of a drainage tube or the presence of a fever before the pleural fluid test between the two groups.

**TABLE 1:** Patient characteristics of pleural fluid culture positive in automated blood culture system by additional positivity of conventional culture (numbers are presented as n (%) or median [interquartile range])^*a*^

Characteristics	Automated blood culture system positive only (*N* = 330)	Both positive (*N* = 302)	*P*-value
Sex			0.060
Male	234 (70.9)	234 (77.5)	
Female	96 (29.1)	68 (22.5)	
Age	65.5 [55.8;73.0]	63.0 [53.0;71.0]	0.016
Pleural fluid			
WBC			<0.001
≤500 (/μL)	60 (19.5)	20 (8.6)	
>500 (/μL)	248 (80.5)	212 (91.4)	
Protein (mg/dL)	3270.0 [2363.3;4278.3]	3255.7 [2127.6;4147.5]	0.005
LDH (IU/L)	876.0 [416.0;2161.0]	3014.0 [1115.0;10979.0]	<0.001
Glucose (mg/dL)	98.0 [45.0;137.0]	7.0 [2.0;71.5]	<0.001
pH	7.3 [7.3;7.4]	7.3 [7.2;7.4]	0.006
Blood			
WBC (/μL)	11900 [7280;17470]	12270 [8350;16690]	0.866
CRP (mg/dL)	11.41 [4.84;19.64]	15.29 [8.11;25.23]	<0.001
LDH (IU/L)	474.0 [318.0;673.0]	428.0 [294.0;628.0]	0.451
Protein (mg/dL)	5660 [5000;6400]	5600 [5000;6300]	0.511
Pleural fluid/blood ratio			
LDH	1.71 [0.74;4.61]	5.55 [1.96;22.68]	<0.001
Protein	0.59 [0.47;0.71]	0.60 [0.40;0.72]	0.799
Pre-existing chest tube			0.132
No	188 (57.0)	154 (51.0)	
Yes	142 (43.0)	148 (49.0)	
Fever (above 38.0°C)			0.608
No	213 (64.5)	189 (62.6)	
Yes	117 (35.5)	113 (37.4)	

^*a*^ WBC, white blood cell; LDH, lactate dehydrogenase; CRP, C-reactive protein.

### Independent factors associated with the identification of microorganisms in ABCS alone

To identify patient groups that might benefit from the use of ABCS, logistic regression analysis was performed. Protein, LDH, glucose levels in pleural fluid, as well as CRP levels in blood, were identified as independent significant factors associated with exclusive positivity in ABCS (Table 2). In individuals having higher glucose levels in pleural fluid along with elevated WBC and lower CRP levels in blood, ABCS is likely to yield better results than conventional culture.

**TABLE 2:** Factors associated with culture positivity only in automated blood culture system in the multiple logistic regression model^*a*^

Characteristic	Odds ratio (95% CI)		*P*-value
Sex			
Male	0.972 (0.545-1.734)		0.923
Female			
Age (years)	1.010 (0.991-1.029)		0.311
Pleural fluid			
WBC			0.068
≤500 (/μL)			
>500 (/μL)	0.464 (0.203-1.059)		
Protein (mg/dL)	1.000 (1.0001-1.0005)		0.009
LDH (U/L)	1.000 (0.9999-1.0000)		<0.001
Glucose (mg/dL)	1.025 (1.003-1.055)		0.001
pH	0.774 (0.216-2.775)		0.694
Blood			
WBC (/μL)	1.025 (1.003-1.011)		0.093
CRP (mg/dL)	0.954 (0.932-0.977)		<0.001
LD (IU/L)	1.000 (0.999-1.001)		0.692
Protein (mg/dL)	1.061 (0.797-1.413)		0.684
Pre-existing chest tube			
No			
Yes	0.868 (0.522-1.444)		0.587
Fever (above 38.0℃)			
No			
Yes	1.231 (0.724-2.093)		0.442

^*a*^ WBC, white blood cell; LDH, lactate dehydrogenase; CRP, C-reactive protein; CI, confidence interval.

A total of 327 patients had pleural fluid pH ≤7.2 or glucose <60 mg/dL and underwent subgroup analyses (Table S2). Although the concordance rate between ABCS and conventional culture improved by 32.9% p (from 30.1% to 63.0%) in this subgroup compared with the overall cohort, microorganisms were identified in about one-third of patients with ABCS only. There were no significant differences in gender or age between the two groups. Among patients meeting the criteria for complicated parapneumonic effusion, pleural fluid glucose was significantly higher in patients with positive ABCS alone. In contrast, pleural LDH and pH levels were significantly lower in patients with positive ABCS alone. However, age, pleural fluid WBC count, protein levels, and blood chemistry showed no significant differences between the two groups.

### Overall bacteriology

When comparing two groups concerning species identification and the number of identified species, the concordance rate between the two methods was 30.1%. Within the conventional culture group, 52.3% showed no microbial growth, 38.4% were monomicrobial, and 9.3% were polymicrobial. In the ABCS group, 80.1% exhibited monomicrobial growth and 19.9% showed polymicrobial growth. In 18 cases (2.8%), conventional culture yielded more microorganisms than ABCS, whereas ABCS either matched (*n* = 192, 30.4%) or yielded more microorganisms (*n* = 422, 66.8%) than conventional culture in the remaining cases.

**Fig 2:**
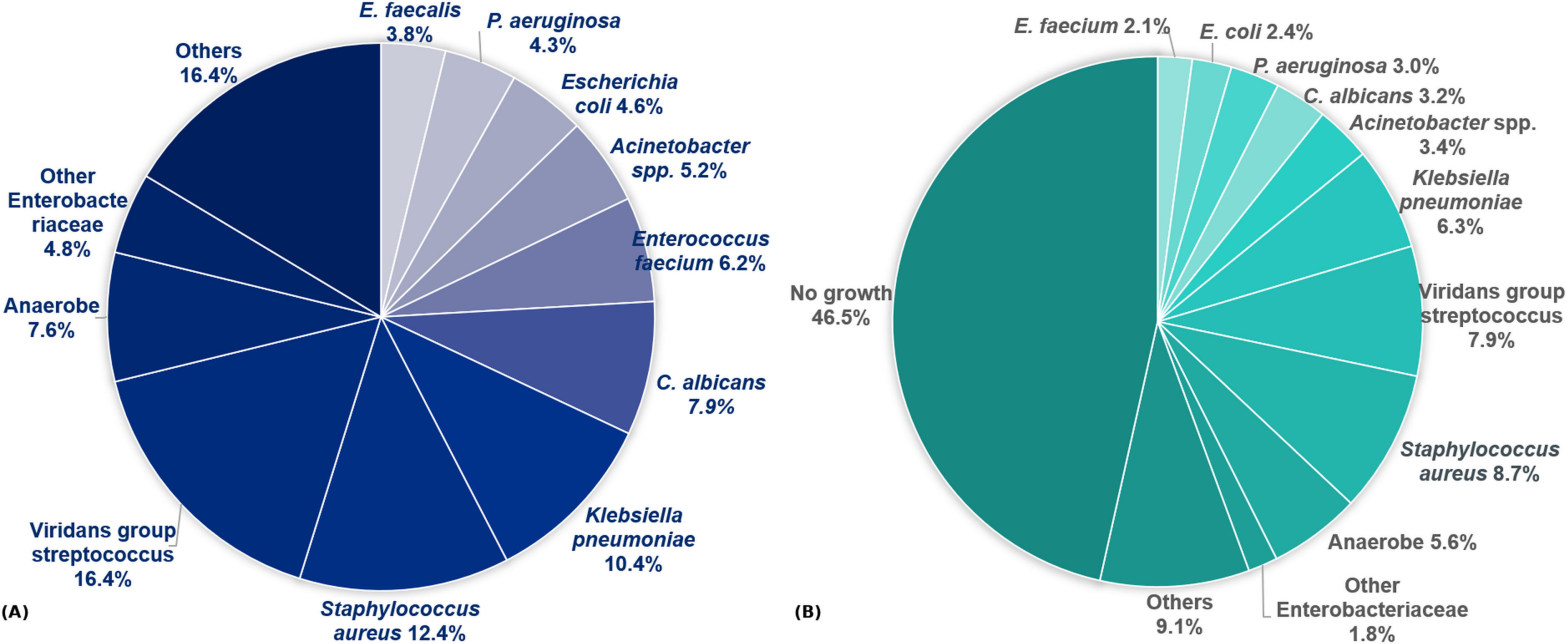
Species distribution according to culture methods. (**A**) Species from automated blood culture system (*N* = 807), (**B**) Species from conventional culture (*N* = 710).

The most frequently isolated bacteria in ABCS were viridans group *streptococcus* (VGS; 16.4%), followed by *Staphylococcus aureus* (12.4%) and *Klebsiella pneumoniae* (10.4%) (Fig. 2A). In conventional culture, the top three species were *S. aureus* (8.7%), viridans group *streptococcus* (7.9%), and *K. pneumoniae* (6.3%) (Fig. 2B).

### Species identified by ABCS alone and their antimicrobial susceptibility

Given that exclusive microbial growth in ABCS can potentially impact treatment decisions, we further analyzed bacteria isolated solely in ABCS. VGS (14.1%) was the most frequent species isolated in ABCS only, followed by *S. aureus* (9.2%), *K. pneumoniae* (7.9%), and *E. faecium* (7.9%) (Tables 3 and 4). Among gram-negative bacteria, *K. pneumoniae* was the most frequently identified organism with 29 cases, followed by *P. aeruginosa* (*n* = 15), *E. coli* (*n* = 15), and *Acinetobacter baumannii* (*n* = 13). Among these, ESCR *K. pneumoniae,* ESCR *E. coli*, CR *P. aeruginosa*, and CR *A. baumannii* accounted for 13.8%, 53.3%, 26.7% (XDR for 13.3%), and 61.5% (XDR for 53.8%) of cases, respectively. Among gram-positive bacteria, methicillin-resistant *S. aureus* (MRSA) and vancomycin-resistant *Enterococcus* (VRE) accounted for 55.9% of *S. aureus* and 30.8% of *Enterococcus*, respectively. In addition, 10% of VGS exhibited resistance to third-generation cephalosporins. Among 368 microorganisms isolated only in ABCS, 56.0% were susceptible to the combination of ceftriaxone and metronidazole, whereas 61.1% demonstrated susceptibility to piperacillin/tazobactam when used as an empirical therapy.

**TABLE 3:** Species distribution of microorganisms isolated with ABCS

Species (*N* = 368)	Number (%)
Viridans group S*treptococcus*	52 (14.1)
*Staphylococcus aureus*	34 (9.2)
*Candida* species	34 (9.2)
*Klebsiella pneumoniae*	32 (8.7)
*Enterococcus faecium*	29 (7.9)
*Pseudomonas aeruginosa*	15 (4.1)
*Escherichia coli*	15 (4.1)
*Acinetobacter* species	13 (3.5)
*Enterococcus faecalis*	12 (3.3)
Other *Enterobacteriaceae*	16 (4.3)
Anaerobe	42 (11.4)
Others	74 (20.2)

**TABLE 4:** Species distribution of microorganisms and antimicrobial susceptibility isolated with ABCS^*a*^^,^^*b*^

	Amp/sul [R]	3rd Cepha [R]	Pip/tazo [R]	Carbapenem [R]	AG [R]	FQ [R]	TMP/XMS [R]
*Klebsiella pneumoniae* (*N* = 29)	3 (10.3)	4 (13.8)	2 (6.9)	1 (4.3)	1 (3.4)	3 (10.3)	3 (10.3)
*Pseudomonas aeruginosa (N = 15)*	15 (100)	4 (26.7)	4 (26.7)	4 (26.7)	2 (13.3)	3 (20.0)	1 (6.7)
*Escherichia coli* (*N* = 15)	5 (33.3)	8 (53.3)	2 (13.3)	0 (0)	0 (0)	8 (53.3)	8 (53.3)
*Acinetobacter baumannii* (*N* = 13)	9 (69.2)	9 (69.2)	8 (61.5)	8 (61.5)	7 (53.8)	8 (61.5)	6 (46.2)
*Stenotrophomonas maltophilia* (*N* = 6)		5 (83.3)				3 (50)	2 (33.3)
*Enterobacter* species (*N* = 6)	6 (100)	3 (50)	2 (33.3)	0 (0)	0 (0)	0 (0)	1 (16.7)
*Serratia marcescens* (*N* = 3)	3 (100)	0 (0)	0 (0)	0 (0)	0 (0)	0 (0)	0 (0)
*Citrobacter* species (*N* = 2)	2 (100)	1 (50)	1 (50)	0 (0)	0 (0)	0 (0)	0 (0)

	Amp [R]	Oxacillin [R]	3rd Cepha [R]	Vancomycin [R]	FQ [R]	Clindamycin [R]	Tetracycline [R]
Viridans group *streptococcus* (*N* = 50)	6 (12.0)		5 (10.0)	0 (0)	2 (4.0)	8 (16.7)	16 (33.3)
*Staphylococcus aureus* (*N* = 34)		19 (55.9)		0 (0)	13 (38.4)	14 (41.2)	12 (35.3)
*Enterococcus faecium* (*N* = 27)	27 (100)			14 (51.9)	27 (100)	27 (100)	4 (14.8)
*Enterococcus faecalis* (*N* = 12)	0 (0)			0 (0)	5 (41.7)	12 (100)	8 (66.7)
*Streptococcus pneumonia* (*N* = 6)			2 (33.3)	0 (0)	0 (0)	4 (66.7)	4 (66.7)

^*a*^ Bacteria for which susceptibility results were not reported were excluded.

^*b*^ Abbreviation: Amp/sul; ampicillin/sulbactam, Amp; ampicillin, R; resistance, Cepha; cephalosporin, Pip/tazo; piperacillin/tazobactam, AG; aminoglycoside, FQ; fluoroquinolone, TMP/SMX; trimethoprim/sulfamethoxazole.

### BACT/ALERT 3D vs VIRTUO

Among the 632 ABCS tests performed, 545 (86.2%) were conducted using the BACT/ALERT 3D, whereas 87 (13.8%) were performed using the BACT/ALERT VIRTUO system (Table S3). The proportion of polymicrobial growth did not differ significantly between the two systems. Although the concordance rate with conventional culture was slightly higher with the VIRTUO (36.8%) than with the 3D (29.0%), this difference was not statistically significant. Furthermore, there was no significant difference in the distribution of microorganisms identified between the two systems, except for *S. aureus*, which showed a significantly higher detection rate with 3D (adjusted *P* = 0.042).

## DISCUSSION

ABCS demonstrated an enhanced diagnostic yield compared with conventional bacterial culture methods. Patients whose microorganisms were identified with ABCS alone had older age, lower serum LDH, higher levels of protein, glucose, and pH in pleural fluid, and lower CRP. Higher glucose levels in pleural fluid, higher blood WBC, and lower CRP were independently associated with cultures being positive only in ABCS.

Conventional culture and ABCS each possess unique characteristics in microbial detection (18). Conventional culture generally uses a smaller inoculum volume and relies on agar-based identification methods, which necessitate additional time for results. This method provides semi-quantitative information based on colony-forming units. In contrast, ABCS allows inoculation of a larger volume of specimens, potentially increasing the likelihood of pathogen detection. It often yields results faster due to real-time monitoring and earlier detection of growth, although it mainly provides qualitative results. However, it is also important to note potential limitations of ABCS: (i) a small number of colonizing microbes may be detected by ABCS, resulting in false positives, and (ii) overgrowth of rapidly growing organisms may obscure the detection of slower-growing species in polymicrobial infections. Despite these limitations, the enhanced diagnostic sensitivity of ABCS has been shown in many studies (10–12).

Results of this study were consistent with those of previous studies. In two previous studies (13, 14), inoculating into blood culture bottles increased the culture positivity rate of pleural infections, with one study showing an increase of 20.8% p (37.7% vs 58.5%) (14). Similarly, in this study, the increased culture positivity rate using ABCS was confirmed to be 34.2% p (31.4% vs 65.6%), slightly higher compared with the previous study. Furthermore, this study found a considerably higher proportion of polymicrobial detection when utilizing ABCS (19.9% vs 9.3%), similar to the results of previous studies (10, 13, 19, 20). Akçam et al. have reported that organisms such as *Brucella melitensis*, *Neisseria meningitidis*, *Pseudomonas fluorescens*, and *Rothia dentocariosa* could be detected only with the BACTEM system (21). Although these organisms were all identified by conventional culture as the same as ABCS in this study, *Aeromonas hydrophila*, *Burkholderia cepacia*, *Chryseobacterium indologenes, Cryptococcus neoformans*, *Cunninghamella bertholletiae*, *Elizabethkingia anophelis*, *Listeria monocytogenes*, *Mycobacterium chelonae*, and non-tuberculous *Mycobacterium* were only detected with ABCS. This study, which included a large number of patients, suggested that ABCS could increase the diagnostic yield of pleural infections, indicating that ABCS should become a part of standard testing.

The higher sensitivity of ABCS may lead to a higher risk of false positives, necessitating subculture and species identification, which consumes staff time and resources. Additionally, there is a risk of unnecessary or inappropriate antibiotic use. However, a total of 180 results in our study were excluded as contaminants, which accounted for only 2% of all screened patients. In contrast, 330 cases were identified exclusively by ABCS and were considered clinically significant, demonstrating that ABCS enhances diagnostic accuracy and may improve the appropriateness of antibiotic treatment. Moreover, among microorganisms identified in ABCS alone, ESCR *E. coli*, CR *A. baumannii*, and MRSA accounted for more than 50%. Given that these microorganisms exhibited resistance to empirical antibiotics such as ceftriaxone and metronidazole or ampicillin/sulbactam and considering that a significant number of multidrug-resistant organisms were detected exclusively through ABCS, this approach could be valuable from a therapeutic standpoint. Thus, the benefit of the higher chance of microbiologic diagnosis and appropriate antimicrobial treatment is likely to outweigh the cost caused by false positive results.

The most frequently isolated bacteria in ABCS were VGS (16.4%), *S. aureus* (12.4%), and *K. pneumoniae* (10.4%), whereas the top three species in conventional culture were *S. aureus* (8.7%), VGS (7.9%), and *K. pneumoniae* (6.3%). These findings slightly differed from a previous study where *S. aureus* (20.7%) was the most frequent aerobic isolate, followed by VGS (18.7%), *Pseudomonas* spp. (17.6%), *Enterobacteriaceae* (11.9%), and *Streptococcus pneumoniae* (10.8%) (7). Even with conventional cultures as a reference, *Pseudomonas* and *S. pneumoniae* were less frequently identified in our study. Community-acquired and hospital-acquired pleural infections were not differentiated in our study. Given that *P. aeruginosa* is a common cause of healthcare-associated infections and that previous studies have frequently isolated *P. aeruginosa* in a hospital-acquired setting (7, 22), a significant proportion of patients included in this study might be in a community-acquired setting. Nevertheless, given the nature of tertiary hospitals where a substantial number of patients undergo repeated hospitalizations and antibiotic treatments, further analysis might be necessary to determine whether many patients are indeed in a true community-acquired setting, or if there are other reasons for the lower incidence of *P. aeruginosa*. Regarding *S. pneumoniae*, the decreasing incidence of invasive pneumococcal disease in Korea following the introduction of vaccination might have accounted for the lower prevalence observed in our study (23).

The laboratory test results of the patients whose pleural fluid cultures were positive only in ABCS were significantly different compared with those of the patients with cultures positive in both methods. Exclusively ABCS-positive patients had a lower proportion of pleural fluid WBC >500/ µL, higher median pleural fluid glucose, lower median LDH, and lower median blood WBC and CRP levels. Those results suggest less severe pleural inflammation, and probably less severe infection, in this group. Lower microbial burden in those patients may have been the cause of negative culture in conventional methods.

BACT/ALERT VIRTUO system has been FDA-approved for sterile body fluids. It is known to offer advantages such as improved automation and faster time to detection, whereas its detection capability has been reported to be equivalent to that of the BACT/ALERT 3D system (24, 25). In this study, we could not assess the time to detection due to the retrospective nature of the study. However, there were no significant differences in other diagnostic characteristics between the two systems, including polymicrobial detection, concordance with conventional culture, and recovery of the major pathogens. Notably, *S. aureus* was recovered more frequently with the BACT/ALERT 3D system.

This study has several limitations. First, as a retrospective study, ABCS was not performed for all patients with pleural infection. Second, due to the nature of the clinical data warehouse, certain variables such as turbidity of pleural fluid, inoculation volume, and time difference in reporting between ABCS and conventional culture could not be collected. Third, this was a single-center study. Given the characteristics of a tertiary care hospital, patients might have had more comorbidities and drainage tubes compared with the general population, which may limit the generalizability of our findings. Finally, although commonly considered contaminants were excluded, we could not exclude the possibility that microorganisms identified in ABCS alone could cause infections.

In conclusion, ABCS demonstrated a higher diagnostic yield than conventional culture, even in cases with a higher suspicion of pleural infections such as CPPE. This study supports more proactive utilization of ABCS as a complementary method to conventional culture, particularly when pleural infection is suspected, to improve microbiological diagnosis and treatment decisions.

## SUPPLEMENTAL MATERIAL

Supplemental tables spectrum.00352-25-s0001.docx Tables S1 to S3.

ASM does not own the copyrights to Supplemental Material that may be linked to, or accessed through, an article. The authors have granted ASM a non-exclusive, world-wide license to publish the Supplemental Material files. Please contact the corresponding author directly for reuse.
