# Supplementary material for: Comparison of conventional culture and automated blood culture system for microbiologic diagnosis of pleural infection
Source: Microbiol Spectr. 2025 Jun 12;13(8):e00352-25. doi: 10.1128/spectrum.00352-25 (PMC12323579; doi:10.1128/spectrum.00352-25)
Supplement: Supplemental tables — Tables S1 to S3. [file spectrum.00352-25-s0001.docx]

**Comparison of Diagnostic Power of Conventional Culture and Automated Blood Culture System with Pleural Fluid: A Retrospective Study**

**SUPPLEMENTARY MATERIALS**

**Table of Contents**

**Table S1.** Characteristics of the patients whose pleural fluid cultures were positive in conventional culture only

**Table S2.** Subgroup analysis of the patients whose pleural fluid pH ≤7.2 or glucose <60 mg/dL

**Table S3**. Comparison of (A) performance and (B) species distribution between BACT/ALERT 3D and VIRTUO.

**Table S1**. Characteristics of the patients whose pleural fluid cultures were positive in conventional culture only (numbers are presented as n (%) or median [interquartile range])

| **Characteristics** | **Conventional culture positive only (N=5)**  **N (%) or median [IQR]** |
| --- | --- |
| Sex |  |
| Male | 4 (80) |
| Female | 1 (20) |
| Age | 66.0 [54.0;77.0] |
| Pleural fluid |  |
| WBC |  |
| ≤500 (/μL) | 1 (20.0) |
| >500 (/μL) | 4 (80.0) |
| Protein (mg/dL) | 5062.4 [4658.6;5173.5] |
| LDH (U/L) | 926.0 [468.0;1384.0] |
| Glucose (mg/dL) | 90.0 [12.0;91.0] |
| pH | 7.4 [7.3;7.4] |
| Blood |  |
| WBC (/μL) | 11620 [11470;21110] |
| CRP (mg/dL) | 8.81 [6.71;23.72] |
| LD (IU/L) | 464.0 [278;547] |
| Protein (mg/dL) | 5570 [5500;5900] |
| Pleural fluid/blood ratio |  |
| LD | 2.00 [1.85;2.53] |
| Protein | 0.88 [0.75;0.91] |
| Pre-existing chest tube |  |
| No | 5 (100) |
| Yes | 0 (0) |
| Fever (above 38.0℃) |  |
| No | 3 (60.0) |
| Yes | 2 (40.0) |
| Species |  |
| Staphylococcus aureus | 2 (40) |
| Viridans streptococcus group | 2 (40) |
| Klebsiella pneumoniae | 1 (20) |

**Table S2**. Subgroup analysis of the patients whose pleural fluid pH ≤7.2 or glucose <60 mg/dL

| **Characteristics** | **Automated blood culture system positive only (N=132)** | **Both positive (N=195)** | **P** |
| --- | --- | --- | --- |
|  | **N (%) or mean ± SD or median [IQR]** | |  |
| Sex |  |  | 0.425 |
| Male | 100 (75.8) | 155 (79.5) |  |
| Female | 32 (24.2) | 40 (20.5) |  |
| Age | 65.0 [55.5;72.0] | 63.0 [53.0;70.0] | 0.152 |
| Pleural fluid |  |  |  |
| WBC |  |  | 0.531 |
| ≤500 (/μL) | 12 (9.2) | 12 (7.2) |  |
| >500 (/μL) | 118 (90.8) | 154 (92.8) |  |
| Protein (mg/dL) | 3860.0 [2580.0;4548.0] | 3410.0 [2188.2;4290.9] | 0.073 |
| LDH (U/L) | 1855.0 [722.8;4967.3] | 3966.0 [1568.5;12606.5] | <0.001 |
| Glucose (mg/dL) | 28.5 [4.0;98.0] | 4.0 [2.0;22.0] | 0.003 |
| pH | 7.2 [7.2;7.4] | 7.3 [7.2;7.4] | <0.001 |
| Blood |  |  |  |
| WBC (/μL) | 13980 [9620;19640] | 12640 [9240;17230] | 0.087 |
| CRP (mg/dL) | 13.46 [6.71;23.72] | 17.71 [9.07;26.99] | 0.073 |
| LD (IU/L) | 458.0 [309.8;614.3] | 420.0 [293.0;596.8] | 0.625 |
| Protein (mg/dL) | 5900 [5200;6500] | 5700 [5100;6375] | 0.054 |
| Pleural fluid/blood ratio |  |  |  |
| LD | 4.10 [1.61;10.00] | 8.51 [3.15;29.56] | <0.001 |
| Protein | 0.65 [0.53;0.74] | 0.63 [0.41;0.72] | 0.552 |
| Pre-existing chest tube |  |  | 0.244 |
| No | 79 (59.8) | 104 (53.3) |  |
| Yes | 53 (40.2) | 91 (46.7) |  |
| Fever (above 38.0℃) |  |  | 0.865 |
| No | 80 (60.6) | 120 (61.5) |  |
| Yes | 52 (39.4) | 75 (38.5) |  |

SD, standard deviation; IQR, interquartile range; WBC, white blood cell; LDH, lactate dehydrogenase; CRP, C-reactive protein

**Table S3**. Comparison of (A) performance and (B) species distribution between BACT/ALERT 3D and VIRTUO.

**(A)**

| **Characteristic** | **BACT/ALERT 3D (N=545)** | **BACT/ALERT VIRTUO (N=87)** | **p-value** |
| --- | --- | --- | --- |
|  | **Number (%)** | |  |
| Number of species |  |  | 0.500 |
| Monomicrobial | 434 (79.6) | 72 (82.8) |  |
| Polymicrobial | 111 (20.4) | 15 (17.2) |  |
| Conventional culture |  |  | 0.720 |
| No growth | 283 (51.9) | 47 (54.0) |  |
| Monomicrobial | 209 (38.3) | 34 (39.1) |  |
| Polymicrobial | 53 (9.7) | 6 (6.9) |  |
| Concordance | 158 (29.0) | 32 (36.8) | 0.141 |

**(B)**

| **Species** | **BACT/ALERT 3D (N=700)** | **BACT/ALERT VIRTUO (N=107)** |
| --- | --- | --- |
|  | **Number (%)** | |
| Viridans group streptococci | 106 (15.1) | 26 (24.3) |
| *Staphylococcus aureus* | 96 (13.7) | 4 (3.7) |
| *Klebsiella pneumoniae* | 77 (11.0) | 7 (6.5) |
| *Candida* species | 55 (7.9) | 9 (8.4) |
| Anaerobes | 52 (7.4) | 9 (8.4) |
| *Enterococcus faecium* | 42 (6.0) | 8 (7.5) |
| *Acinetobacter* species | 32 (5.4) | 4 (3.7) |
| *Escherichia coli* | 29 (4.1) | 8 (7.5) |
| *Enterococcus faecalis* | 28 (4.0) | 3 (2.8) |
| *Pseudomonas aeruginosa* | 25 (3.6) | 10 (9.3) |
| Coagulase negative staphylococci | 14 (2.0) | 0 (0) |
| *Streptococcus pneumoniae* | 12 (1.7) | 1 (0.9) |
| Others | 132 (18.1) | 18 (17.0) |
